# Aural Catarrh, or Otitis Media

**Published:** 1870-07

**Authors:** 


### AURAL CATARRH, or OTITIS MEDIA.

Every physician is aware of the great frequency of catarrhal affections of the nasal and pharyngeal mucous membrane in our northern climate; and those who have given this subject thought, must have noticed how often patients suffering from these troubles are subject to ear ache, noises in the head, and hardness of hearing, on every slight exacerbation of their disease, caused by change of weather or imprudent exposure; yet there are many who are unacquainted with the true pathology of these troubles of the ear, and do not know that there is any good method for their relief at the hand of the general practitioner. The mucous membrane of the Eustachian tube and cavity of the tympanum (or middle ear) is but a continuation of that of the pharynx, therefore it is not strange that an inflammation of the latter should often extend to the former; and it is the aim of this article to show that when the disease is thus extended, or even when the aural mucous membrane is inflamed *per se*, it can be as successfully treated by practitioners unacquainted with the special therapeutics of diseases of the ear, as can inflammations of the mucous membrane in other parts of the system.

Aural catarrh is usually considered of two forms, simple or mucous, and purulent; these may each be subdivided into acute and chronic varieties.

Acute mucous catarrh of the ear occurs more frequently during the changeable weather of spring and autumn, and can usually be traced to some definite cause, frequently a severe "cold;" sometimes the extension of a syphilitic eruption from the mucous membrane of the pharynx, which may occur at any time during the course of the chronic variety of this form of the disease. Usually only one ear is affected to any great extent. The hearing is much impaired; tinnitus aurium is complained of, and there is often severe pain at the commencement of the attack, continuing for several days, with marked eracerbations at nights, causing so much irritation and loss of sleep that the patient runs down rapidly. The pain is not increased by pulling upon the auricle, or by pressure in front of the ear, but may be enhanced by swallowing, or by any sudden movement of the head. In severe cases the mastoid process is sensitive to the touch; the molars are painful; pain extends to the frontal sinus, and sometimes over the whole side of the head; then vertigo is frequent, febrile symptoms more or less prominent, sometimes amounting to delirium, and the patient's countenance usually bears an expression of anxiety. The external auditory canal is scarcely changed; the membrana tympani is of a brilliant red, but does not appear infiltrated and thickened, as when that membrane is the starting point of an inflammation.

In the chronic variety of the mucous form, the symptoms are not so prominent; the disease is known more by its consequence, and it is usually impossible for the patient to state at what time his troubles commenced. The impairment of hearing is gradual; there is no pain, and tinnitus is not always present. Such cases do not often come under the eye of the physician till the disease is far advanced, and the hearing much impaired; then, on examination, we almost invariably find symptoms of naso-pharyrgeal catarrh. The rhinoscope shows the mucous membrane of the pharyrgeal space and orifices of the Eustachian tubes in a state of chronic inflammation. More or less, pathological changes are found in the membrana tympani, such as thickening, opacity, abnormal concavity, and sometimes fatty degeneration, similar to the arcus senilis of the cornea. Writers speak of this variety of the disease being sometimes confined to the Eustachian tube (tubal catarrh), and again to the tympanic cavity, but such a differential diagnosis is hard to make; still we may conclude that when the patient complains of sudden changes occuring in the power of hearing, the affection is more in the tube than in the tympanum, and *vice versa* when a certain amount of deafness is continuous. Very many patients, supposed to be suffering from nervous deafness, are found on examination to have pharyrgeal catarrh, and changes in the membrana tympani point strongly to inflammatory troubles of the tympanic cavity; while a watch, or vibrating tuning fork, placed upon the vertex is easily heard, the sounds being conducted to the labyrinth through the cranial bones, thus proving that the function of the auditory nerve is not destroyed. But cases are not always so insidious; their symptoms vary in intensity, and are sometimes sufficiently clear to form a separate or subacute variety.

Acute purulent aural catarrh is more frequent in childhood than in adult age; it occurs as a concomitant and sequel of the different exanthemata; also with small pox, typhus fever, and tuberculosis, and sometimes as an exacerbation of the chronic variety. The symptoms are similar to those of acute mucous catarrh, only more severe; and the general health of the patient is more disturbed. Fever, vertigo, nausea, delirium, and even stupor are not unfrequently present; and when occuring in the course of febrile diseases are too often referred to the general condition, while the local difficulty is wholly overlooked, till irreparable damage has been done. When we take into account these peculiar and severe symptoms, it is not surprising that acute otitis media is often confounded with meningitis and abcess of the brain, and in the more severe cases, unless relief is speedily afforded, these latter diseases may supervene, and the patient is said to die from the original fever having "gone to the head," and the intermediate tympanic inflammation, and the relief which might at that time have been afforded is entirely ignored. The common result of this purulent inflammation, and to the casual observer the first intimation of the real nature of the disease, is perforation of the membrana tympani, when a discharge of puss takes place, and all the symptoms—with the exception of the deafness—are greatly alleviated. The objective appearances of the external ear are different from acute mucous cases; the membrana tympani—before perforation, is more convex, more swollen and infiltrated; extravasations of blood are not unfrequent upon it, and the inflammation sometimes extends to the external canal, causing a concomitant purulent otitis externa.

As a proof of the frequency of this trouble in infancy, reference may be given to recent *post mortem* examinations made by Dr. Wreden, of St. Petersburgh, on eighty infants, varying in age from a few hours to fourteen months. Out of these one hundred and sixty ears examined, all but fourteen had undergone marked pathological change; and forty-five per cent of the whole number of children were affected with purulent inflammation of the middle ear, yet the membrana tympani was found perforated only once, which shows, dependent as we are entirely on objective symptoms with patients of such tender age, how great the difficulty is in reaching an exact diagnosis; yet were it possible to always judge correctly on this point, how many a child might be kept from asylums for the education of deaf mutes.

Chronic purulent aural catarrh is more frequent than the acute variety; it may occur without any previous acute inflammation, but more frequently is an extension from inflammation of the membrana tympani, or an otitis externa, or it is a sequel of the acute variety last described. Suppurative inflammation of the middle ear cannot be long continued without the destruction of a greater or less portion of the membrana tympani; consequently a purulent discharge from the external meatus is always present during the course of this variety of the disease; this discharge, and the impairment of hearing, are the only constant symptoms. The opening through the drum, if small, cannot always be seen, but after thoroughly cleansing the external canal, if we examine the membrane while air is forced into the tympanic cavity through the Eustachian tube, some of the purulent secretion will be seen oozing through the perforation, and a whistling noise will be heard. If a large portion of the membrane be destroyed, some parts of the tympanic cavity may be seen by using a strong light, reflected from a concave mirror of about six inches focal distance.

The prognosis in cases of aural catarrh depends much upon the amount of pathological change that has taken place in the parts. In acute mucous cases it may be said to be favorable, with this exception, that there often remains a strong predisposition to a relapse, which may finally end in the continuous chronic variety; more especially is this true in our Northern and Eastern States, where the changeable climate acts as a strong exciting cause in those cases subject to the above predisposition.

The chronic mucous variety may also be looked upon favorably when seen at an early date; and even where the inflammation has been long continued there are very many cases in which, by appropriate treatment, the inflammatory process may be stopped, and the hearing more or less benefited. In both of the above varieties there is a tendency to thickening of the mucous membrane, and synechial adhesions between the different parts of the tympanum, as between the different bones of the ossicula, between each of these and the tympanic walls, between the membrana tympani and the promontory, and a pseudo anchylosis between the stapes and the membrane of the fenestra ovalis; it will readily be perceived that such adhesions would prove a source of irritation, as well here as when occuring in the pleura, pericadium, or on the iris; and as they would also limit the vibrations of the membranes and ossicula, must interfere materially with the conduction of sounds to the labrynth. Fortunately in some cases these adhesions are not so strong but what they may be broken up or loosened by the use of the air bath. The thickening of this mucous membrane of the Eustachian tube may cause its closure, the air in the tympanum then becoming rarefied by absorption, the membrana tympani concave by atmospheric pressure from without, the normal functions of the ear would thus be interfered with, unless the closed tube can again be opened to the passage of air. If the Eustachian tubes are easily permeable to air, which may be ascertained by Polityus method—soon to be described—or by the Valaavan experiment of making a strong expiration, with the mouth and nostrils shut, the chances of successful treatment are better than when those tubes seem closed.

The prospect of a favorable termination in the purulent form of the disease is not as good as in the mucous, but in the majority of acute cases it would be much better, if physicians would pay the least attention to the ear in those febrile disorders that usually give rise to this variety; if properly and quickly treated, the greater number of such cases will recover, without any noticeable impairment of the hearing.

But in the chronic variety, where the patient has been afflicted for a long time with a purulent otorrhœa, we can never be sure how much benefit we may be able to give, for we cannot tell precisely the extent of injury; and our opinion must be held in abeyance till the result of treatment is ascertained. Every inflammation of the mucous membrane of the middle ear is also a periostitis, for the aural mucous membrane cannot be separated or distinguished from the periostium. The cavity of the middle ear is separated from the meninges of the cerebrum above, and from the internal jugular vein below, by plates of bone, so thin that in many skulls they are translucent; its direct communication with the internal ear and auditory nerve is cut off only by the delicate membranes of the fenestra rotunda and ovalis; nor are the internal carotid artery and facial nerve much better protected; is it then surprising that a long-continued inflammation of the tympanum, producing as it sometimes does caries of the long walls of that cavity, should occasionally be followed by meningitis, cerebral abcess, phlebitis, pyaemia, fatal hemorrhage, and facial paralysis; any one or several of these may be the sequel of a case of otorrhœa, and many such cases have been reported within the last five years, yet how often do intelligent physicians reply to an anxious parent's enquiries in regard such a discharge, "let it alone, he will outgrow it." Would any surgeon give such advice in a case of suppuration from caries of the parietal bone or the tibia? Yet the latter might with much more safety be let alone. Fortunately, we find by applying the test of treatment to these chronic cases that the most of them may be benefited, in very many, the inflammation and discharge cured, and a useful amount of hearing be preserved.

In the treatment of aural catarrh, I propose to dwell only on those methods that may be used by the general practitioner, without his possessing that skill and experience necessary to the specialist; and it is but recently that such medication has been possible. In all the different varieties of this disease, the inflation of the tympanic cavity with air through the Eustachion tubes, or air bath as it is called, has long been considered a necessity, but as the only thorough method of doing this was by use of the Eustachian catheter; and as the majority of physicians are unacquainted with the use of that instrument, such cases were not often benefited, unless they fell into the hands of some well-informed aurist. But Politzer, of Vienna, in 1863, gave a great boon to humanity by the suggestion of a method of accomplishing this inflation, so simple that even children may be taught its use. It had been proved by experiment previous to that time, that during the act of deglutition the Eustachian tube was drawn open by the action of the tensor palati muscle; taking advantage of this fact, all that was necessary to inflate the tympanum was to force air into the nasal cavity at the same instant that the patient swallowed, with the mouth and nostrils closed; the Eustachian tubes being open, and the air having no other chance of egress, would naturally pass through and fill the tympanic cavities; and that it does so is known by the patient experiencing a sensation of fullness in the ears. Some improvements have been made on the instrument first used by Politzer. The one now most frequently used is a rubber bag, connected by a tube with a glass nozzel, made to fit the nostril, and large enough to contain a sponge, on which tincture of iodine, or other volatile medicines may be placed, and their vapors carried into the ears with the air.

In the acute mucous variety the air bath should be used as soon as possible, that the Eustachian tube may be opened, and exit given to a part at least of the secretions that are distending the tympanum. The frequency of its use should depend upon the severity of the symptoms. I have used it from one to three times a day. Local blood letting, frequently filling the ear with warm water, and diaphoretics, are of great benefit in easing the pain and subduing the inflammation; and the hypodermic injection of $\frac{1}{4}$ grain of morphia, with $\frac{1}{60}$ grain of sulphate of atropia, I have found to be of much benefit in the severe cases. The following case is a good example of this variety, and in it, as well as others that I shall cite, no treatment was used requiring special skill.

Mr. E. ——, aged 58, Sept. 30th, 1859. Has severe pain, extending from right ear over whole side of head; has some tinnitis aurium; it hurts him to move the jaws, and is almost impossible to swallow; had not been able to sleep during the previous night. The day before had been exposed to a severe storm, and thoroughly drenched, wearing his wet clothes for some time. Previous to this attack had never had trouble with the ears, or been subject to catarrhal symptoms of any kind. The membrana tympani is inflamed and brilliant; the pharynx shows no symptoms of chronic trouble, but is inflamed and swollen on the right. Could only hear the ticking of watch on right side, when pressed against the ear, while on the left it could be heard at the distance of four feet. Used Politzer's bag, carefully, which gave him some relief. Ordered three leeches to the tragus, the ear to be frequently filled with warm water, and gave hypodermic injection of morphiæ and atropiœ at night.

Oct. 1st. Passed an uncomfortable night, but did not have so much pain as the night before. Air bath used three times this day; perspiration induced by two five-drop doses of tinc aconite; warm water continued, and subcutaneous injection repeated. After this he suffered no severe pain, and the hearing gradually improved under the use of the air bath till the 8th, when, on account of careless exposure, pain and febrile symptoms returned; the leeches and other treatment were again used, which gave him speedy relief, and he continued to improve.

Nov. 15th. No tinnitis aurium; membrana tympani normal; hears watch on right side at arm's length as distinctly as on left.

In chronic mucous cases the air bath should always be employed. Politzer's bag, with the addition of a few drops of tincture of iodine or acetic acid, may be used two or three times a week, or even daily, according to the severity of the symptoms; and in the early history of cases, counter irritation to the mastoid process may be of use, but can do little good when the disease has been long continued. Patients should be cautioned against the use of ear drops, poultices, and the introduction of instruments of any kind into the external meatus. The naso-pharyngeal mucous membrane should receive strict attention, as in this class of cases it is almost always diseased, and is usually the nidus of the affection, so that what ever good we do by local treatment of the ear is sure to be of short duration if the mucous membrane of the pharynx and mouths of the Eustachian tubes are left in a state of chronic congestion. This may be treated by Weber's nasal douche;* or if the patient is frequently seen by the physician, by the naso-pharyngeal syringe using stimulating or astringent washes, as the case may need. Gargles of alum, or of iodine, to which a little brandy has been added, are of much use; and if the tonsils or uvula are hypertrophied, they should be excised. The general health of the patient should receive careful attention; strict hygienic rules should be enforced, and a wholesome dietic regimen established. General tonics are often necessary, and many cases are benefited by mild alteratives, not too long continued. Cases with syphilitic bases, while undergoing an appropriate constitutional course, should still receive local treatment.

Anna H ——, aged 7, July 9th, 1867. Has had trouble in hearing for the last year; is worse when suffering from cold; complains of noises in the head, but no pain; general condition good; does not readily hear when spoken to; hears watch on right side of the head six inches from auricle, on left only three. The drums are more brilliant than natural, especially the left; no change in their curvature; mucous membrane of pharynx slightly congested; Politzer's

*Although the wisdom of using the nasal douche has been recently questioned by several good authorities,—vide Knapp's, Archives, and Transactions of the American Otoligical Society, for 1869, it being claimed that supperative inflammation of the tympani cavity is some times caused by the introduction of fluids through the Eustachian tubes, I am still of opinion that this danger might be guarded against, and the douche safely used by giving the patient the following directions. viz.: First—hold the head low, and bend forward, letting the chin rest upon the chest; this will take a great part of the weight of the fluid in the nasal cavity off from the soft palate, so that it will not be so liable to bend down, letting the fluid into the throat and causing a disposition to swallow. Second—If the fluid does get into the throat, and the patient feels that he must swallow, have him take the nozzle of the douche instantly from the nostril, and the effort at swallowing will at once cease. Third—Do not allow the patient to use a fluid that is not warmer than the surrounding atmosphere. I think it better to have it near the bodily temperature, and do not use a solution sufficiently irritating to cause smarting or pain in the Schnaderian membrane. By attention to the first and second of these directions it is almost an impossibility for the fluid to reach the tympanni, especially if it is conceded that the Eustachian tubes are only open during the act of deglutition, as seems to be reaffirmed by Voltotine's recent experiment in a pneumatic cabinet; and by the third the possibility of its doing harm in case it did pass through the tubes is rendered nil. My own experience in something over 200 cases is that mild solutions are more efficacious than those which irritate, and I have never had so much as an ear ache follow the use of the douche, the use of which seems to me a much more thorough means of applying solutions to the nasal mucous membrane than by the syringe. I would not wish to be understood as thinking it a safe instrument to be sold by druggists and venders of patent medicines to the people, but when properly used, under the direction of a physician, it seems too valuable to be thrown aside.

method, with tincture of iodine, was used every other day; a gargle of iodine ordered, and the naso-pharyngeal syringe, with cleansing solution of pumanganate of potash used six times during the treatment, which was continued till August 5th, when her hearing appeared normal, and she complained no more of tinnitus.

Chas. L——, aged 19, June 12th, 1868. Has been hard of hearing for the last eight years, during all of which time he has been slowly growing worse. Has never suffered ear ache, but has constant noises in the head. Has had no discharge from the ears. General health not good; is of an evident scrofulous diathesis. Does not hear ordinary conversation. Right ear hears watch only when pressed, the left at two inches from auricle. Both drums seem thickened, opaque, and slightly concave. Mucous membrane of pharynx is granulated, and secretes excessively. Used Politzer's bag, with tincture of iodine, every other day; Weber's douche, with solution of carbolic acid and iodine daily; to use iodine gargle, and take hypophosphites; also gave strict rules for diet and hygiene.

July 14th. Continued the above treatment up to this time. Pharyngeal catarrh much better; hypersecretion almost entirely stopped; can hear ordinary conversation, and the watch four inches from the right ear, and fourteen inches from the left. General health improved. As the patient resided some distance in the country, he was sent home with directions to continue the treatment, with the exception of the gargle and douche, which were only to be used when there were symptoms of pharyngeal catarrh. I saw him about a month since. During the last year he has had several exacerbations of his catarrhal trouble, and at those times would be hard of hearing; but, by the prompt use of the air bath and douche, they were quickly alleviated. His general health was better, and he retained the amount of improvement in hearing narrated above.

These two cases show the necessity of early treatment in this insidious variety of aural catarrh, where every "cold in the head," if left to itself, serves to thicken the aural mucous membrane, and increase the deafness. The first case, treated one year after apparent commencement of disease, was easily cured; the second, allowed its own course for eight years, was only susceptible of improvement.

The treatment of acute purulent aural catarrh must be strongly antiphlogistic, local blood-letting, to a greater or less extent, according to the condition of the patient; the ear frequently filled with warm water; attention given to the condition of the bowels, and diaphoretics employed when febrile symptoms demand. Politzer's bag should be used, being careful not to exert too much force; and the mucous membrane of the pharynx must receive attention, especially where the otitis has occurred in consequence of one of the exanthemata. Gargles should be used frequently, the posterior nerves cleaned by injections, and sometimes the application of argenti nitras, xx gr. to the oz., is of much benefit when the pharyngeal mucous membrane is inclined to ulceration. Narcotics will afford some relief, but cannot be relied upon as curative. In the case of young children simultaneous swallowing is not essential to the success of Politzer's method, which should be used with care. The nasal cavity should be kept clear by syringing, or by provoking sneezing by the introduction of an oiled feather through the nostril into the pharynx. The induction of vomiting may sometimes be of use; and if the child is robust, the application of one or two leeches to the tragus will be of benefit. But very great relief may be given in those cases where the air bath fails to evacuate the pus through the Eustachian tube, by paracentesis of the membrana tympani; and as in these cases, perforation of that membrane is sure to occur if left to itself, it is as justifiable to anticipate as it would be in the case of any inflammatory abscess; and by giving early exit to the pus, we prevent much of that softening and change in the parts that would take place if left alone. The paracentesis may be done with a cataract needle or exploring trocar, after which the air bath should be used.

A journeyman printer came to me, March 24th, 1869, complaining of severe pain in the left ear; it had continued for the last 48 hours; he had had no sleep, and some of the time had been delirious; he could not walk without assistance, and his countenance was expressive of the utmost anxiety. Opiates had been administered, and poultices and leeches applied, without relief. Previous to this attack he had never had pain in the ears, but had suffered from naso pharyngeal catarrh, noises in the head, and been hard of hearing for several years. Could not hear watch on left side when pressed against the ear, and only four inches distant on the right.

Right drum opaque and somewhat sunken; the left, convex, swollen and infiltrated, while the inner half of the external meatus was inflamed; pharynx inflamed and secreting profusely. Made an incision about a line in length through the most prominent part of drum, and used Politzer's bag; quite a discharge of pus occurred, and the severe pain was relieved. Used the air bath and nasal douche twice a day; gave an iodine gargle, and a carbolic acid wash for external ear.

March 26th. Opening through the drum closed; no severe pain; continued treatment.

April 1st. Much better; no pain, tinnitis slight; hears easily when spoken to; hears watch on right side eight inches from ear, on left three; condition of pharynx improved; ordered to contiune air bath and douche daily while troublesome symptoms last.

Oct. 20th. Patient called at my request for an examination. Says he has not been so free from catarrh, or heard so well, for the last five years, as during the past summer. Hearing distance of watch on right side twenty-four inches; on left side ten. There is no apparent scar on membrana tympani left by the paracentesis.

Hattie M——, aged 8, April 21st, 1868. Has been suffering from scarlatina for the last ten days. Naso-pharyngeal mucous membrane inflamed, excoriating and discharging a thick muco purulent secretion. For the last twenty-four hours has complained of her ears and head paining her, and hearing queer noises. Does not hear unless spoken to loudly; can distinguish the tick of a watch one inch from right ear and two from the left. Drums are both inflamed, but do not appear prominent. Used naso-pharyngeal syringe first, with carbolic solution, then with argenti nitras, gr. iv. to the oz., and Politzer's bag twice a day; ordered three leeches to each tragus; gave alum gargle and hypodermic injection of $\frac{1}{8}$ gr. of morphia at night.

April 22nd. Passed a comfortable night; does not complain of so much pain to-day; continued treatment.

April 24th. Much better. Changed the washes to iodine, to be used once a day, air bath the same.

June 19th. Patient well; pharyngeal mucous membrane and hearing normal. The severity of the symptoms in this case, and the ease with which they were combated, can but favor the impression that many of our patients, who date their deafness and troublesome otorrhœa from an exanthematous attack, might have been saved all such danger and annoyance had Politzer's method and the concomi treatment been in general use.

The great desideratum in the treatment of chronic purulent variety is cleanliness; and did this object receive proper attention from the commencement of the accompanying otorrhœa, I think the terrible sequala that have been previously noticed would be entirely avoided. The careful injection of tepid water, to which a little carbolic acid has been added, I have found the most serviceable as a cleanser and deodorizer. When the use of the syringe is not well borne a camel's hair pencil may be substituted. This cleansing should be repeated two or three times a day, if necessary, to keep the ear free from secretions. If the perforation through the drum be small, the air bath will assist in cleansing the tympanum and forcing the secretion into the external meatus. After the ear is cleansed, a solution of sulphate of zinc, from i. to iv. gr. to the oz., may be dropped into the ear, or sulphate of copper, or acetate of lead, in equal parts, with acetic acid may be used. If the head is held to one side, so as to keep the drops from running out, and while in that position the air bath is used, it will facilitate the introduction of the solution into the tympanum. If the discharge is kept up by a polypus it should be removed, not by tortion, for fear of injuring surrounding parts, but by Wilde's polypus snare, which acts upon the same principle as the ecraseur. After the otorrhœa has stopped, if the perforation through the membrana tympani does not seem inclined to close, the hearing may be benefited in many cases by wèaring an artificial drum. The same attention to the general health of the patient should be given as in the other forms of the disease.

Willie H.——, aged 9, June 6th, 1867. Had scarlatina three years since. During convalescence had pain and a discharge from both ears; the latter has continued more or less profuse since. His general health is fair. Does not hear watch on right side when pressed against the ear or the bones of the head, but can hear it one inch from left ear. Both Eustachian tubes are pervious. Right membrana tympani almost entirely destroyed; malleus and nicus gone, but stapis visible; mucous membrane of tympanic cavity thickened; left membrana tympani has small central perforation, through which a yellow puriform discharge exudes on the use of the air bath. Gave him Politzer's bag, to be used twice a day, followed by a cleansing wash, and astringent drops for the external meatus.

July 17th. Ottorrhœa nearly cured. Hears watch on left side three inches, and on the introduction of artificial drum twelve inches; but no improvement was produced on right side, even with the drum.

W. A.———, aged 19, Sept. 23rd, 1868. Twelve years ago, had measles, which left his ears in a bad condition; the right discharged for a long time, but has not troubled him for the past few years with the exception that he does not hear in it. Hears watch on right side only when pressed against the ear; on left at a distance of four feet. Right membrana tympani has small perforation, through which air can be blown with Politzer's bag. The left is normal. On placing artificial drum in right ear could hear the watch fifteen inches distant. The first of these patients wore the artificial drum without much benefit; the other had no occasion for doing so, as the hearing was normal in the left ear; but should that ear become deaf in the future, the use of the drum in the right would be of great service.

Having given a rèsumè of the treatment that will prove beneficial in a great majority of cases of aural catarrh, allow me to say in conclusion that should these remedial agents fail to produce a cure, on account of impermeability of the Eustachean tube, cleft or ulcerative destruction of the palate, or old pathological changes in the tympanic cavity, there is still hope that the patient may be benefited, as there are methods other than those just detailed by which such cases may be treated, such as the Eustachian catheter, by means of which, not only the air bath may be used more thoroughly, even than by Politzer's method, but gases and fluids may be introduced into the tympanum, and bougies, or probes, used to overcome strictures and dilate the Eustachian tube. The Eustachian nebulizer may sometimes be of benefit. Operative procedures on the membrani tympani, and, ossicula, to break up adhesions can do no harm in those cases where hearing has been for some time lost, and they have, in some instances, proved of benefit.

AUTHORITIES.

Much of the literature of this subject is only accessible to those conversant with the German, but the following recent publications in English may be consulted with benefit:

Allen, Peter, M. D., F.R.C.S., on some of the Functions of the Middle and Internal Ear and their analogies. London, Lancet, 1869.

Anderson, Dr. T. Mc All, on Politezr's Method of Inflating the Eustachian Tube. Glasgow Medical Journal, 1866.

Archives, of Ophthalmology and Otolygy. New York, 1869.

Beard, George M., M.D. The Membrana Tympani and Pharynx in Deaf Mutism. American Journal of Medical Science, April, 1867.

Bishop, Edward, M.D., M.R.C.S.E., New Method of Applying Remedial Agents to the Cavity of the Tympanum. Pamphlet. London, 1866.

Hinton, James, Clinical Remarks on Perforation of the Membrana Tympani. Guy's Hospital Reports, vol. xii., 1866.

Politzer, on the Membrana Tympani; translated by Drs. Matthewson and Newton. Just issued.

Rossa, D. B. St. John, M.D. Statistical Report of Five Hundred cases of Aural Disease. New York Medical Journal, August, 1869. On Advancement in Diagnosis and Treament of Diseases of the Ear. Transactions of New York State Medical Society, 1868. On the Membrana Tympani in Deaf Mutes. American Journal of Medical Sciences, April, 1867. On Catarrhal Inflammation of the Middle Ear occuring in Young Persons. American Journal of Medical Sciences, July, 1865.

Transactions of the American Otological Society, 1869.

Trôltsh, on the Ear. Translated by Dr. Rossa, 1869.

